# Late-Onset Puerperal Psychosis: A Case Report and Challenges in Treatment Decisions

**DOI:** 10.1192/j.eurpsy.2025.2412

**Published:** 2025-08-26

**Authors:** P. Ibáñez Mendoza, C. Delicado Gascon, B. Orgaz Álvarez, M. Velasco Santos, Á. de Vicente Blanco

**Affiliations:** 1 Psychiatry, Hospital Universitario La Paz, Madrid, Spain

## Abstract

**Introduction:**

The perinatal period is a vulnerable time for women, with specific risk factors for mental health issues. Puerperal psychosis typically presents within the first month postpartum, although the perinatal period extends through the first year after delivery. This condition is understudied, and its nature and pathophysiology remain subjects of debate.

**Objectives:**

To describe a case of late-onset puerperal psychosis, highlighting the challenges in decision-making regarding medical approach.

**Methods:**

A clinical case report and a non-systematic review of the literature.

**Results:**

A 39-year-old woman was brought to the Emergency Unit by her relatives due to paranoid delusions that her partner and in-laws were attempting to poison her. She had previously sought mental health care only once, as an adolescent, for anxiety symptoms following her parents’ divorce. She is the mother of a 5-year-old child and a 10-month-old infant, with no reported complications during pregnancy or delivery.

The patient reported experiencing strange occurrences over the preceding 10 days, beginning during a family vacation when she became suspicious of the food and the organization of meals. She believed her in-laws were poisoning her and expressed concern about transmitting poison to her infant through breast milk. Upon returning home, these fears intensified, extending to suspicions that her husband, mother, and sister were involved. She had drastically reduced her food intake the prior days before consulting, her appearance was malnourished and disheveled.

Further psychopathological exploration revealed delusional beliefs centered on being poisoned and potentially poisoning her baby through breastfeeding. These delusions were accompanied by confusion, perplexity, and heightened anxiety. She denied experiencing hallucinations and had no thoughts of harm toward herself or others.

Low-dose olanzapine treatment was initiated and outpatient management was initially chosen to minimize disruption to her role as a mother, in accordance with the patient’s preference and the presence of family support. However, hospitalization ultimately became necessary, resulting in complete resolution of psychotic symptoms after 14 days, with olanzapine titrated to a higher dose (20 mg per day).

**Conclusions:**

Puerperal psychosis is a complex condition with potentially severe consequences for both maternal and infant health, including disruptions in mother-child bonding. This case underscores the need for further research and resource allocation in this area. Specifically, the development of more psychiatric mother-baby units could help prevent unnecessary separations, promote bonding, and provide opportunities for early parenting interventions.

**Disclosure of Interest:**

None Declared

